# Tasmanian devil whale optimization (TDWO) is introduced for secure video transmission in 5G networks

**DOI:** 10.1371/journal.pone.0330270

**Published:** 2025-08-18

**Authors:** Fengren Lin, Minrong Lu

**Affiliations:** 1 Department of Information Engineering, Fuzhou Polytechnic, Fuzhou, China; 2 Department of Accountancy, Fujian Jiangxia University, Fuzhou, China; Northwestern Polytechnical University, CHINA

## Abstract

With the extensive growth of the web as well as cellular networks, secure multimedia transmission through cellular networks is needed. Currently, fifth-generation (5G) cellular networks are utilized to perform secure multimedia transmission. Numerous studies have been conducted to design efficient resource allocation approaches for secure video transmission in 5G cellular networks. However, this approach does not offer complete video security related to security against dynamic eavesdroppers or patent defilements. Thus, a resource allocation algorithm named Tasmanian devil whale optimization (TDWO) is introduced for secure video transmission in 5G networks. Here, the recorded educational videos are considered and are transmitted over 5G network transmission resources initially. The resources in 5G networks are allocated via the TDWO model by considering fitness parameters such as the data rate, achievable data rate, and quality of experience (QoE). Here, the deep convolutional neural network (DCNN) model is deployed for the prediction of the QoE in resource allocation. Moreover, extensive experiments are performed to identify the resource allocation performance of the designed TDWO model. The experimental results prove that the TDWO resource allocation algorithm yields significant experimental outcomes, with throughput, bit error rate (BER), QoE and fitness values of 25.557 Mbps, 0.021, 18.332 and 0.013, respectively.

## 1 Introduction

In recent years, the rapid expansion of video and multimedia services over wireless networks has dramatically increased the demand for advanced telecommunication technologies, particularly fifth-generation (5G) networks [1]. 5G’s high data capacity and ultra-low latency have created a critical infrastructure for applications such as remote education, video conferencing, and telemedicine [2,3]. One of the main challenges is to design resource allocation algorithms that not only ensure security and energy efficiency but also maximize user Quality of Experience (QoE) under the strict requirements of these modern applications [4]. Adaptive Resource Allocation (ARA) algorithms are among the most widely used techniques, utilizing instantaneous channel state information and user requirements to dynamically distribute resources, thereby enhancing spectral efficiency and reducing interference [5]. Nevertheless, these methods often struggle to guarantee multi-objective QoS, exhibiting high packet loss rates and reduced system performance when handling dynamic traffic loads or multiple concurrent services [6,7]. Martin et al. [8] demonstrated that resource allocation based solely on network conditions often fails to provide a truly optimized end-user experience.

In recent years, Software-Defined Networking (SDN) and Network Function Virtualization (NFV) technologies have introduced unprecedented flexibility into network resource management [9]. These frameworks automate network control and can rapidly adapt to fluctuating user demands, thereby improving efficiency [10]. However, studies reveal that many SDN/NFV-based solutions neglect crucial QoE factors, such as quality switching events and end-to-end latency, which are essential for interactive and high-quality video services [11,12]. Specialized video encoding and lightweight encryption methods such as HEVC and advanced coding algorithms have also contributed to volatile improvements in video data security and computational overhead reduction [13]. For example, Thiyagarajan et al. [14] demonstrated the effectiveness of these schemes for IoT video transmissions, while other works have noted that most encoding-focused solutions apply only at the application or coding layers, rarely considering their interaction with energy consumption or network layer QoE [15].

Device-to-Device (D2D) communication models, which leverage direct user pairing and resource allocation, have shown significant promise for scalable video delivery [16,17]. However, improper or suboptimal pairing due to limited resources or increased traffic congestion can undermine overall system performance [18]. Research indicates that D2D solutions are most effective in scenarios where resources are plentiful and network conditions are relatively stable [19]. Recently, bio-inspired metaheuristic algorithms such as Genetic Algorithm (GA), Particle Swarm Optimization (PSO), Whale Optimization Algorithm (WOA), and the newly introduced Tasmanian Devil Optimization (TDO) have emerged as powerful tools for tackling complex resource allocation problems in 5G networks [20,21]. Thanks to their global search capabilities and robustness against local minima, these techniques are well-suited for dynamic, multi-objective optimization in modern mobile systems [22]. Studies by Dehghani et al. [23] and Mirjalili et al. [24] have emphasized the advantages of hybrid metaheuristics in accelerating convergence and improving overall solution robustness. A major limitation of conventional techniques lies in the lack of a deep understanding of how technical performance metrics relate to perceived user experience [25]. Statistical analyses suggest that solutions focusing exclusively on spectrum efficiency or energy savings cannot guarantee satisfactory user experiences across challenging conditions [26]. As a result, the integration of Deep Learning—particularly with Deep Convolutional Neural Networks (DCNN)—and metaheuristic optimization has become a popular direction in next-generation resource management research [27,28].

Models leveraging DCNNs for QoE prediction are able to model the intricate relationships between user assignment, channel status, and subjective perception, thus bridging the gap between network-level decisions and end-user satisfaction [29]. Recent works demonstrate that the combination of Deep Learning with metaheuristic algorithms can simultaneously improve security, spectral efficiency, and QoE—critical for educational video streaming where high content quality and continuity are paramount [30,31]. This is especially important in the context of educational services, where poor video quality or interruptions can significantly affect learning outcomes [32].

To address the above limitations, this paper proposes a new hybrid algorithm called **Tasmanian Devil Whale Optimization (TDWO)**, which combines elements of bio-inspired optimization with deep learning and multi-objective fitness functions [33]. By integrating the exploratory power of TDO and the fast exploitation ability of WOA, TDWO achieves a superior trade-off between solution diversity and convergence speed in 5G resource allocation [34]. Critically, the incorporation of a DCNN-based QoE predictor ensures that the allocation decisions dynamically reflect both technical and user-centric performance goals [35]. Experimental results confirm that the TDWO algorithm consistently outperforms traditional methods—such as ARA, SDN/NFV-based resource allocators, HEVC, D2D matching, and stand-alone metaheuristics—across all major metrics, including system throughput, BER, and user-perceived QoE [8,23,32]. These innovations solidify TDWO as a potent solution for secure, high-quality video delivery over 5G, with potential transformative benefits for education and other multimedia-driven domains.

## 2 Research methods

While video transmission is a cornerstone of 5G network development—playing a vital role in advancing education, business, and healthcare through applications like distance conferencing and e-learning—existing techniques often overlook differentiated resource allocation, highlighting the need for an optimization-based deep learning model to securely and flexibly allocate resources for video transmission in 5G networks.

### 2.1 Current problems

Despite advancements in methods for secure video transmission and resource allocation—such as adaptive resource allocation (ARA), network resource allocators leveraging SDN/NFV, chaos-based encryption, high-efficiency video coding (HEVC), noise aggregation models, security protocols for multimedia, and D2D association frameworks—existing schemes suffer from drawbacks including high packet loss, insufficient QoE consideration, limited real-time performance, security vulnerabilities, inflexible resource allocation, and suboptimal trade-offs between efficiency and service quality, underscoring the ongoing need for optimization-based deep learning approaches tailored for 5G educational video transmission.

### 2.2 System model of the educational video network

**Fig 1:**
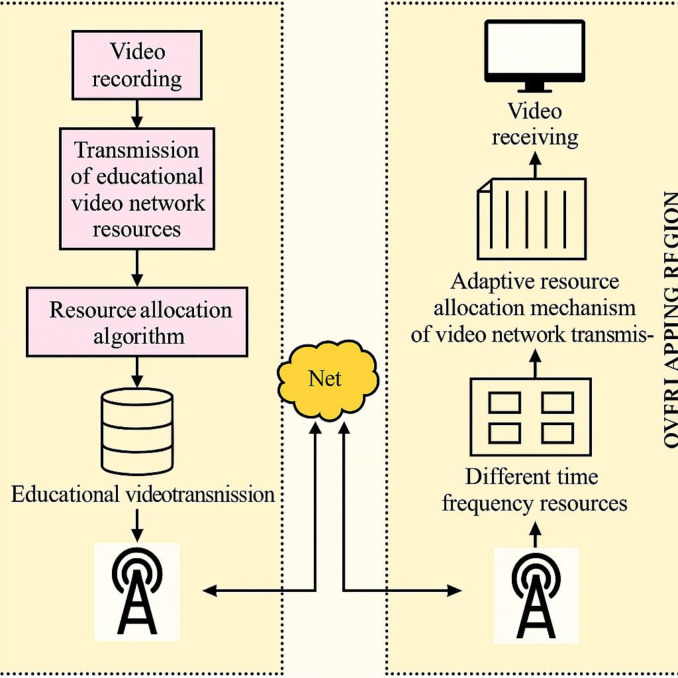
System model of the educational video network.

In the proposed system, efficient resource allocation across both normal and overlapping regions utilizes time-frequency resources to optimize single-frequency network transmission, minimize co-frequency interference, and conserve spectrum by employing advanced algorithms and adaptive mechanisms; within a downlink orthogonal frequency network of g g g cells (frequency reuse factor 1), educational video services are transmitted over defined propagation areas—comprised of specific cell groupings, with overlapping cells accommodating both unicast (from the serving cell) and multicast (from the propagation area) transmission modes, as illustrated in Fig 1.

This figure illustrates the overall system model for the educational video network, focusing on resource allocation and network architecture in the context of 5G. The system model likely depicts a cellular network layout with multiple cells (propagation areas) where educational video services are transmitted. The figure should show multiple cells representing different geographical areas covered by the network.The diagram should highlight overlapping regions between cells, indicating areas where users might receive signals from multiple base stations. The text mentions cell ‘c’ as an overlapping area. Base stations within each cell should be represented, indicating the source of the video transmission.Users receiving unicast and multicast services within the network. The text specifies a frequency reuse factor of 1, meaning each cell uses the same frequency band. The figure might visually represent this. The model shows users receiving both unicast (dedicated) and multicast (shared) video streams. The purpose of this figure is to show the physical layout of the network and how resources are shared or allocated within this structure.

### 2.3 Channel model and transmission structure

Consider a downlink system supporting W W W user terminals, where a base station equipped with A A A transmitting antennas and each user terminal with a single receiving antenna transmits over a frequency band divided into δ \delta δ subcarriers (assuming W ≥ A W \geq A W ≥ A); within each channel’s coherent interval, the channel matrix remains constant, and the received signal for user c c c on the l l l-th subcarrier is expressed as follows.


$$R_{z,l}=G_{c,l}S_{l}+D_{c,l}$$ (1)

Here, the gain matrix of the channel of the user c is represented as $G_{c.l}$, which comprises the same unit variance as the independent mean. The transmitted symbol for the antenna base station is $S_{l}$, and the complex Gaussian noise is $D_{c.l}$.

In equation (1) have:

- The signal received by user *u* on subcarrier *k*.
- The gain matrix of the channel of user *u*. It characterizes the communication channel between the base station and user *u*, including factors like path loss, fading, and interference.
- The transmitted symbol for the antenna base station. Represents the signal that the base station sends.
- Complex Gaussian noise. Represents the background noise and interference in the communication channel.
- This equation models the received signal as a function of the channel characteristics, the transmitted signal, and the noise.

A zero-forcing beam-forming transmission approach is employed at the transmitting end for the selection of active user groups, and the values of the data to be transmitted are set to $I_{l}$. Here, the beamforming vector $L_{c,l}$ is multiplied by the data symbol $I_{c,l}$ and is given by the expression


$$S_{l}=\sum_{c\in L_{l}}\;\;\sqrt{F_{c,l}}I_{c,l}L_{c,l}$$ (2)

- The beamforming vector. Used to focus the transmitted signal towards specific users.
- Data to be transmitted.
- Power allocated to user for transmission.
- This equation demonstrates how the data symbol is multiplied by the beamforming vector and power allocation factor to create a focused transmission signal.

where F indicates the power allocated to the user for transmission. Thus, equation (1) is given by


$$R_{z,l}=G_{z,l}\sum_{c\in L_{l}}\;\;\sqrt{F_{c,l}}I_{c,l}L_{c,l}+D_{z,l}$$ (3)

- This equation substitutes equation (2) into equation (1), showing the received signal incorporating the beamforming vector.

If $c\neq zG_{z.l}\gamma_{z.l}=0$, the zero interference condition is achieved by the beamforming vector, and the corresponding values of the submatrix are $K_{z}\left(L_{z}\right)$ and $I_{z}\left(L_{z}\right)$. Moreover, the pseudoinverse of $K_{z}\left(L_{z}\right)$ is performed to simply obtain the beamforming matrix and is designated as


$$I_{z}\left(L_{z}\right)=K_{z}{\left(L_{z}\right)}^{+}=K_{z}\left(L_{z}\right)*{\left(K_{z}\left(L_{z}\right)K_{z}{\left(L_{z}\right)}^{*}\right)}^{-1}$$ (4)

This defines how to calculate the beamforming matrix using the pseudoinverse of the channel matrix.

### 2.4 TDWO design for resource allocation in 5G networks

**Fig 2:**
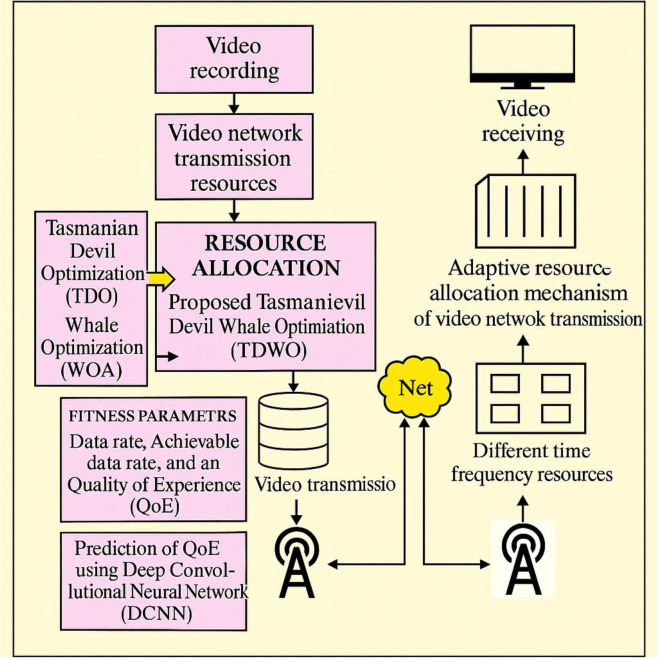
Diagrammatic view of TDWO for resource allocation in a 5G network.

The primary purpose of the TDWO model is to achieve efficient resource allocation for secure video transmission in 5G networks by transmitting recorded video from the database, employing the integrated TDO [22] and WOA [23] algorithmic approaches to optimize data rate and QoE as fitness parameters, and utilizing the DCNN [24] model for QoE prediction, as illustrated in Fig 2.

This figure provides a high-level overview of the TDWO algorithm’s workflow for resource allocation in a 5G network. The diagram should illustrate the major steps involved in the TDWO process. The process starts with “Recorded video acquisition” of educational videos, likely taken from a database. The core of the diagram should illustrate the TDWO algorithm itself, showing how it combines elements of both the Tasmanian Devil Optimization (TDO) and Whale Optimization Algorithm (WOA).The figure should show that the TDWO algorithm considers fitness parameters such as data rate, achievable data rate, and Quality of Experience (QoE). A key element is the integration of the Deep Convolutional Neural Network (DCNN) for QoE prediction. The figure should show the DCNN receiving some input (likely related to network conditions or user assignments) and outputting a QoE prediction. This predicted QoE is fed back into the TDWO algorithm as part of the fitness evaluation. The final output of the TDWO process is the allocation of resources in the 5G network to ensure secure and efficient video transmission. The purpose of this figure is to provide a clear, visual understanding of how the TDWO algorithm works and how it integrates with the DCNN for QoE prediction.

#### 2.4.1 Recorded video acquisition.

The input recorded educational video considered for secure video transmission in 5G networks by allocating resources is taken from an Alankar Kotwal implementation database [25] and is expressed as


$$V=\left\{V_{1},V_{2},\ldots ,V_{P}\ldots V_{X}\right\}$$ (5)

Here, the database taken for video transmission in 5G networks is denoted as V, the $P^{th}$ video recording considered for secure transmission is represented as $V_{P}$, and the total number of video recordings available in the dataset is X.

- The normalized achievable data rate of user *u* on subcarrier *k*.
- The power of the subcarrier *k*.
- Calculates the achievable data rate for a specific user on a specific subcarrier, considering the power allocated to that subcarrier.

#### 2.4.2 Video transmission.

Video transmission is regarded as a typical large-capacity application, enabling the delivery of both low-stream and high-resolution videos. Network transmission resources are leveraged to enhance the quality and reliability of video content. Furthermore, improving the quality of educational video transmission increases the system’s transmission rate and enables greater diversity gains. In this context, the recorded video VP V_P VP is transmitted over 5G cellular networks to deliver high-quality educational videos, thereby enhancing security, throughput, coverage, and bandwidth.

#### 2.4.3 Adaptive resource allocation algorithm.

After educational videos are transmitted to the 5G network, an optimized resource allocation algorithm is applied to efficiently assign spectral, temporal, spatial, and power resources, ensuring secure and reliable video transmission while meeting network demands; the detailed resource allocation process is described below.

(1) **Achievable data rate**

The total number of bits that can be securely transmitted in a given channel per second achievable data rate is termed the achievable data rate [26], which is responsible for the speed of video transmission in 5G networks and is considered during resource allocation in 5G cellular networks. Let us assume that the multiplex is permitted by M≥2 users and that the user cluster considered for subcarrier f is $\beta_{f}=\left[x_{1},x_{2},x_{3},\ldots ,x_{n}\right]$, where the user cluster $x_{1},x_{2},x_{3},\ldots ,x_{n}\in \{1,2,3,\ldots ,O\}$, where O refers to the number of users. Thus, the normalized achievable data rate of the user $x_{1}$ is given by


$$a_{\beta_{f}}^{f}\left(x_{1}\right)=\log_{2}\left\{1+b_{\beta_{f}}^{f}\left(x_{1}\right)x_{x_{1}}^{f}\right\rfloor$$ (6)

Similar to equation (5), but defines it specifically for user u.

where the power of the subcarrier f is expressed as $b_{\beta_{f}}^{f}$. Similarly, the normalized achievable data rate of user $x_{2}$ is designated as


$$a_{\beta_{f}}^{f}\left(x_{2}\right)=\log_{2}\left[1+\frac{b_{\beta_{f}}^{f}\left(x_{2}\right)x_{x_{2}}^{f}}{1+b_{\beta_{f}}^{f}\left(x_{1}\right)x_{x_{1}}^{f}}\right]$$ (7)

Extends the concept to all subcarriers.

Thus, other users $\beta_{f}$ with a normalized achievable data rate are estimated, and the normalized achievable data rate of all subcarriers f is expressed as


$$A_{SC}^{f}=a_{\beta_{f}}^{f}\left(x_{1}\right)+a_{\beta_{f}}^{f}\left(x_{2}\right)+\ldots .+a_{\beta_{f}}^{f}\left(x_{n}\right)$$ (8)

where the special cases $x_{x_{1}}^{f}=x_{x_{2}}^{f}=\ldots =x_{x_{n}}^{f}$ hold by all the equations during the estimation of the normalized achievable data rate.

- Transmission power of the base station.
- A constant parameter.
- Subcarrier assignment.
- Another constant parameter.
- This equation estimates the transmission power of the base station based on constant parameters and subcarrier assignment.

(2) **Power consumption**

Generally, 5G cellular networks consume more power than other networks do because of the utilization of more frequency bands and network traffic issues. The transmission power of 5 G cellular networks is computed by taking the sum of the static circuit power and the dynamic amplifier power while considering constant parameters $J_{\beta_{f}}^{f}$ and subcarrier assignment $E_{\beta_{f}}^{f}$. The transmission power of the base station is estimated via the expression


$$\psi_{P}=\sum_{x_{1}=1}^{M}\;\;\sum_{x_{2}=1}^{M}\;\;\ldots \sum_{x_{n}=1}^{M}\;\;\sum_{f=1}^{N}\;\;J_{\beta_{f}}^{f}E_{\beta_{f}}^{f}\left(b_{\beta_{f}}^{f}\left(x_{1}\right)+b_{\beta_{f}}^{f}\left(x_{2}\right)+\ldots +b_{\beta_{f}}^{f}\left(x_{n}\right)\right)$$ (9)

- Total power consumed.
- Static circuit power of the base station.
- Amplifier efficiency.
- Estimates the total power consumption based on the static circuit power and amplifier efficiency.

The total power consumed is estimated via the expression


$$\psi =\psi_{c}+v\psi_{P}$$ (10)

- Normalized system throughput over bandwidth.
- This introduces the concept of a normalized system throughput, related to spectral efficiency.

where the static circuit power of the BS is expressed as $\psi_{c}$ and where ν represents the amplifier efficiency:

(3) **Objective model**

In general, users should achieve better trade-offs between spectral efficiency and energy efficiency during resource allocation by considering all available degrees of freedom, such as user clustering, power allocation, subcarrier assignment, and the deployment of multiple access nodes. By effectively balancing spectral and energy efficiency, the total power consumption can be reduced while simultaneously increasing spectral efficiency, as expressed by the following equation.


$$\min_{J,b}\;-\tau (J,b)$$ (11)

where τ signifies the normalized system throughput over bandwidth, b indicates the unit energy, and the binary variable is denoted as J.

- Unit energy.
- Binary variable.
- This provides an expression related to energy efficiency and a binary variable.

$$\min_{J,b}\;P(J,b)$$ (12)

- Minimum one-user cluster adopted by the subcarrier *k*.
- Requirement of the minimum rate guaranteed by the QoS for every user.
- Specifies the constraints for resource allocation, ensuring a minimum number of users per subcarrier and a minimum data rate per user.

which is given by


$$v_{1}\;\;:\psi_{P}\leq \psi_{p}$$ (13)

$$v_{2}\;\;:b_{\beta_{f}}^{f}\left(x_{1}\right)\geq 0,b_{\beta_{f}}^{f}\left(x_{2}\right)\geq 0,\ldots ,b_{\beta_{f}}^{f}\left(x_{n}\right)\geq 0\forall f$$ (14)

$$v_{3}\;\;:\sum_{x_{2}=1}^{M}\;\;\sum_{x_{2}=1}^{M}\;\;\cdots \sum_{x_{n}=1}^{M}\;\;J_{\beta_{f}}^{f}\leq 1\forall f$$ (15)

$$v_{4}\;\;:J_{\beta_{f}}^{f}\in \{0,1\},\leq 1\forall f$$ (16)

$$v_{5}\;\;:\tau_{x}\geq \tau_{\min},\forall x$$ (17)

Equations (13) to (17) respectively define the TDWO fitness function as a weighted combination of achievable data rate, data rate, and DCNN-predicted QoE; introduce and formulate the convolutional and fully connected layers in DCNN for QoE prediction; and mathematically model the Tasmanian devil population for optimization, while constraints v3v_3v3 and v4v_4v4 ensure at least one-user cluster per subcarrier except for the maximum user, and v5v_5v5 enforces the minimum QoS-guaranteed rate for each user.

(4) **Resource allocation**

**Fig 3:**
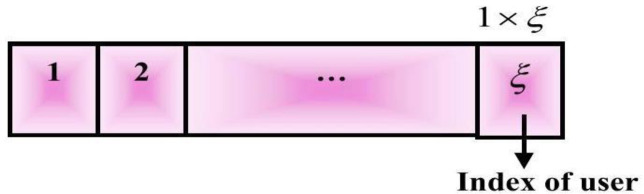
Solution encoding for resource allocation in 5G networks.

Resource allocation in 5G cellular networks facilitates the assignment of network resources to various services and devices, thereby improving spectrum utilization and reducing transmission latency. In this study, resource allocation is conducted using the TDWO approach, which is developed by integrating the Tasmanian Devil Optimization (TDO) [22] and Whale Optimization Algorithm (WOA) [23]. The resource allocation procedure is conducted as follows:

a) Solution encoding:

The resources are allocated in 5G networks by considering the user index of size 1×ξ, where the term ξ signifies total users. The solution is initially zed randomly in 5G networks during the allocation of resources, and Fig 3 depicts the solution encoding performed for the allocation of resources.

This figure explains how potential solutions for resource allocation are encoded within the TDWO algorithm. The solution encoding represents how the TDWO algorithm represents different possible resource allocations. This is crucial for the algorithm’s ability to explore different solutions. The figure should illustrate how user indices are represented. The text mentions a user index of size ‘N’, where N is the total number of users. It needs to show how these user indices are mapped to network resources (e.g., subcarriers, resource blocks, power levels). The solution encoding might be a vector or matrix where each element represents the resource assigned to a particular user. The text mentions that the solution is initially randomized, so the figure might show a random arrangement of user indices mapped to resources. The figure’s goal is to show the structure of a single ‘solution’ that the TDWO algorithm manipulates and optimizes.

b) Fitness computation:

The fitness parameters, such as the data rate, achievable data rate, and QoE, are considered for the allocation of resources in 5G networks. The fitness is estimated via the following formula:


$$\text{Fitness\textasciitilde{}=}\frac{1}{3}\sum_{e=1}^{\xi }\;\;[{\left(A_{SC}^{f}\right)}_{e}+DR_{e}+\sigma_{e}\backslash$$ (18)

where $A_{SC}^{f}$ signifies the achievable data rate, DR indicates the data rate, and $\sigma_{e}$ is the QoE predicted via the DCNN model. The prediction process performed in the DCNN is described below.

Carrion (Equation 18):

- the Tasmanian devil.
- selected carrion by the Tasmanian devil.
- This equation shows that the Tasmanian devils usually feed on carrion.

c) Architecture of the DCNN

The DCNN [24] is a straightforward and efficient model utilized for predicting the Quality of Experience (QoE) during fitness value estimation. In this approach, the user assignment matrix is input into the DCNN to predict the QoE. The DCNN architecture comprises three primary layers: the convolutional layer, the pooling layer, and the fully connected layer. The specific functions of each of these layers are described as follows:

**① Convolutional layer**

The convolutional layer is the most critical component of the DCNN, consisting of multiple consecutively connected layers designed to enhance prediction accuracy. The input data is passed through the convolutional layer, where neurons are utilized to extract more localized features. A feature map is then produced by convolving the input with trainable weights, while a nonlinear activation function processes the output from the preceding convolutional layer. Therefore, the convolutional layer can be mathematically expressed as follows:


$$Y=\left\{Y_{1},Y_{2},\ldots ,Y_{H},\ldots ,Y_{Z}\right\}$$ (19)

Tasmanian Devil Position (Equation 19):

- Tasmanian devil position at the iteration.
- modified position of the Tasmanian devil.
- value of the objective function of the selected carrion
- random number set to the interval
- symbolizes a random number fixed to 1 or 2.
- This equation shows that the modified position of the Tasmanian devil is based on the value of the objective function of the selected carrion.

where Z signifies the total number of convolutional layers that act as inputs to generate outputs, and the output produced by the centered unit (m,n) in the conv layers is expressed as


$${\left(i_{b}^{a}\right)}_{m,n}={\left(j_{b}^{a}\right)}_{m,n}+\sum_{D=1}^{p_{1}^{k-1}}\;\;\sum_{E=-q_{1}^{k}}^{q_{1}^{k}}\;\;\sum_{F=q_{2}^{k}}^{q_{2}^{k}}\;\;{\left(\kappa_{a,D}^{k}\right)}_{E,F}*{\left(i_{b}^{a-1}\right)}_{m+E,n+F}$$ (20)

Modified Tasmanian Devil Position (Equation 20):

- equation (25) is given by this equation.

where the kernel function of the convolutional layer is represented as $\kappa_{a,D}^{k},{\left(i_{b}^{a}\right)}_{m,n}$ indicates a fixed feature map from the convolutional layer and where ${\left(i_{b}^{a-1}\right)}_{m+E,n+F}$ represents the feature map corresponding to the preceding layer of the $b^{\text{th\textasciitilde{}}}$ convolutional layer. The convolutional operation executed by local patterns is denoted as *, and $j_{b}^{a}$ denotes the bias of the $b^{th}$ convolutional layer. Moreover, the activation function is employed in the convolutional layer to identify the output

**②** **Pooling layer**

The global or local pooling layers are employed in convolutional networks to simplify the underlying computation. The dimension of the data is reduced by integrating the neuron cluster outputs from one layer with the single neuron of the next layer. It acts as a subsampling layer that helps minimize the feature map resolutions to increase the feature invariance. As the pooling layer is nonparameterized, it does not have trainable bias or weights.

**③** **Fully connected layer**

The resulting features of the convolutional layer are fed into fully connected layers for the prediction of the QoE, and the output of the fully connected layer is given by the expression


$$U_{b}^{a}=\mu \left(\lambda_{b}^{a}\right)$$ (21)

$$\lambda_{b}^{a}=\sum_{D=1}^{p_{1}^{k-1}}\;\;\sum_{E=-q_{1}^{k}}^{q_{1}^{k}}\;\;\sum_{F=q_{2}^{k}}^{q_{2}^{k}}\;\;{\left(\kappa_{a,D}^{k}\right)}_{E,F}*{\left(i_{b}^{a-1}\right)}_{m+E,n+F}$$ (22)

Best Solution (Equation 21):

- best solution obtained at the iteration.
- coefficient vector
- This equation models the best solution obtained at the iteration, where is the coefficient vector.

**Fig 4:**
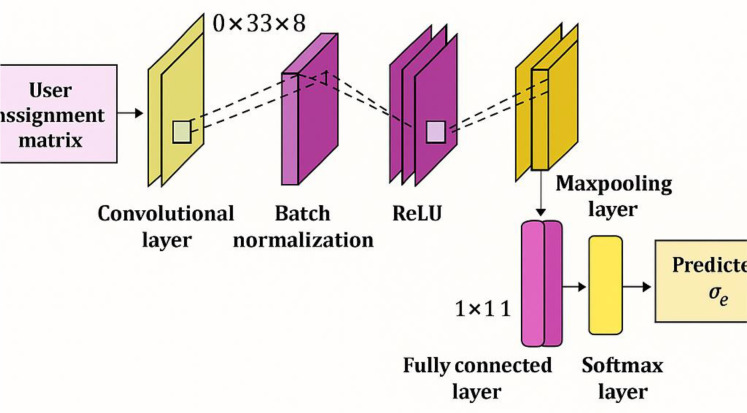
Architecture of the DCNN.

Coefficient Vector (Equation 22):

- equation (29) becomes.

where the weight linking the centered unit (m,n) in the $D^{\text{th\textasciitilde{}}}$ feature map is expressed as $\kappa_{a,D,E,F}^{k}$. Thus, the final output obtained from the fully connected layer is the predicted $QoE\sigma_{e}$, and the architecture of the DCNN is portrayed in Fig 4.

This figure details the structure of the Deep Convolutional Neural Network (DCNN) used for predicting QoE. The DCNN architecture diagram should show the different layers of the network. The text mentions three main layer types: The figure should show one or more convolutional layers. Each layer will have a set of filters (kernels) that convolve with the input to extract features. Pooling layers (e.g., max pooling or average pooling) are used to reduce the dimensionality of the feature maps and make the network more robust to variations in the input. These layers connect every neuron in the previous layer to every neuron in the current layer. They are used to make the final prediction of QoE. The figure should indicate the input to the DCNN (likely the user assignment matrix, as mentioned earlier) and the output, which is the predicted QoE value. The figure is intended to provide a clear understanding of the DCNN’s internal structure and how it processes information to predict QoE.

d) Resource allocation via the TDWO model

Resource allocation in 5G cellular networks is performed using the TDWO algorithm, which is developed by integrating the Tasmanian Devil Optimization (TDO) [22] and the Whale Optimization Algorithm (WOA) [23]. The TDO is inspired by the feeding and behavioral characteristics of Tasmanian devils, which primarily utilize two main strategies: scavenging carrion and hunting live prey. TDO demonstrates strong exploration capabilities for identifying optimal regions and effectively addresses computationally complex problems within the search space. Similarly, the WOA is inspired by the hunting methods of humpback whales, among the most intelligent animals, known for their unique bubble-net feeding technique. In this technique, humpback whales create spirals of bubbles to trap and capture prey. The main stages of WOA include searching for prey, executing the spiral bubble-net feeding maneuver, and encircling the prey. WOA efficiently approximates the global optimum by employing multiple search agents and effectively avoids getting trapped in local optima. By integrating TDO with WOA, ineffective solutions in the search space are eliminated. The mathematical modeling of TDWO is presented below:

**① Initialization**

At the initial stage, the search agent’s population is randomly generated on the basis of the problem constraints. Here, the total variables of the problem are the same as the total elements of each vector population and are expressed as


$$z=[\begin{array}{c} z_{1}\\ 0\\ z_{r}\\ 0\\ z_{P} \end{array}]P\times k=[\begin{array}{ccccc} Z_{1,1} & 0 & Z_{1,v} & 0 & Z_{1,f}\\ 0 & 0 & 0 & 0 & 0\\ Z_{u,1} & 0 & Z_{u,v} & 0 & Z_{u,f}\\ 0 & 0 & 0 & 0 & 0\\ Z_{W,1} & 0 & Z_{W,v} & 0 & Z_{W,f} \end{array}]W\times f$$ (21)

where z signifies the Tasmanian devil population, $Z_{u}$ denotes the $u^{\text{th\textasciitilde{}}}$ candidate solution, $Z_{u,v}$ represents the candidate value of the $v^{\text{th\textasciitilde{}}}$ variable, W indicates the total searching Tasmanian devils, and f symbolizes the total number of variables.

New Tasmanian Devil Position (Equation 23):

- New position of the Tasmania devil
- This equation shows the final updated equation of TDWO.

**②** **Fitness estimation**

The value of fitness is computed via the expression of fitness given in equation (18).

**③ Exploration phase**

In the exploration phase, the Tasmanian devils generally feed on carrion rather than hunting. The characteristics of the Tasmanian devil during the determination of carrion are mathematically designated as


$$P_{C}=Q_{g},C=1,2,\ldots ,\varpi g\in \{1,2,\ldots ,\varpi |g\neq C\}$$ (24)

Here, the Tasmanian devil population is represented as Q, where ϖ indicates the total searching Tasmanian devil, and where $P_{C}$ signifies the selected carrion by the $C^{\text{th\textasciitilde{}}}$ Tasmanian devil.

Prey (Equation 24):

- equation (38) becomes.
- Prey selected by the Tasmanian devil.
- This equation models the prey selected by the Tasmanian devil.

According to the selected carrion, the position of the Tasmanian devil is modified in the search space. In motion simulation of the Tasmanian devil, if the value of the objective function is better, then the Tasmanian devil moves toward the carrion; otherwise, it moves away from the carrion. The movement strategy of the Tasmanian devil is designated as


$$Z_{u,v}(l+1)=\left\{ \begin{array}{cc} Z_{u,v}(l)+R\cdot \left(Q_{u,v}-\alpha \cdot Z_{u,v}(l)\right), & X_{P_{c}}<X_{u};\\ Z_{u,v}(l)+R\cdot \left(Z_{u,v}(l)-Q_{u,v}\right), & \text{\textasciitilde{}Otherwise,\textasciitilde{}} \end{array}\right.$$ (25)

where $Z_{u,v}(l)$ signifies the Tasmanian devil position at the $l^{th}$ iteration, $Z_{u,v}(l+1)$ represents the modified $(l+1{)}^{th}$ position of the Tasmanian devil, the value of the objective function of the selected carrion is denoted as $X_{P_{C}}$ via equation (18), R signifies the random number set to the interval [0,1], and α symbolizes a random number fixed to 1 or 2.

Modified Tasmanian Devil Position Exploitation Phase (Equation 25):

- the position of the Tasmanian devil is modified to improve the target function value once the position of the prey is determined
- This equation models the position of the Tasmanian devil

Considering that $X_{P_{C}}<X_{u}$, equation (25) is given by


$$Z_{u,v}(l+1)=Z_{u,v}(l)+R\cdot \left(Q_{u,v}-\alpha \cdot Z_{u,v}(l)\right)$$ (26)

$$Z_{u,v}(l+1)=Z_{u,v}(l)+R\cdot Q_{u,v}-R\cdot \alpha \cdot Z_{u,v}(l)$$ (27)

$$Z_{u,v}(l+1)=Z_{u,v}(l)[1-R\cdot \alpha ]+R\cdot Q_{u,v}$$ (28)

Equations (26), (27), and (28) respectively describe: the iteration-based process in which the Tasmanian devil’s position progressively approaches the neighborhood center during the chase, the modeling of its updated position through neighborhood interaction, and the throughput, defined as the ratio of successfully transmitted educational videos to the execution time.

The WOA [23] is integrated with TDO [22] for the elimination of ineffective solutions available in the search space. From the WOA,


$$Z_{u,v}(l+1)=Z_{\text{best\textasciitilde{}}}(l)-N\cdot \eta$$ (29)

BER (Equation 29):

- Ratio of total received bits in error to the total transferred bits

Here, the best solution obtained at the l iteration is represented as $Z_{\text{best\textasciitilde{}}}(l)$, the coefficient vector is N, and η denotes the modified position of the best search agent.


$$\eta =|\chi \cdot Z_{\text{best\textasciitilde{}}}(l)-Z_{u,v}(l),Z_{\text{best\textasciitilde{}}}(l)>Z_{u,v}(l)$$ (30)

Thus, equation (29) becomes


$$Z_{u,v}(l+1)=Z_{\text{best\textasciitilde{}}}(l)-N\cdot [\chi \cdot Z_{\text{best\textasciitilde{}}}(l)-Z_{u,v}(l)\backslash$$ (31)

QoE (Equation 31):

- The weight preferred by the client.
- QoE of the video.
- This equation describes how QoE is computed, considering client preferences and video quality.

where χ signifies the coefficient vector.


$$Z_{u,v}(l+1)=Z_{\text{best\textasciitilde{}}}(l)-N\cdot \chi \cdot Z_{\text{best\textasciitilde{}}}(l)+N\cdot Z_{u,v}(l)$$ (32)

$$N\cdot Z_{u,v}(l)=Z_{u,v}(l+1)-Z_{\text{best\textasciitilde{}}}(l)+N\cdot \chi \cdot Z_{\text{best\textasciitilde{}}}(l)$$ (33)

$$N\cdot Z_{u,v}(l)=Z_{u,v}(l+1)-Z_{\text{best\textasciitilde{}}}(l)[1-N\cdot \chi$$ (34)

$$Z_{u,v}(l)=\frac{Z_{u,v}(l+1)-Z_{\text{best\textasciitilde{}}}(l)[1-N\cdot \chi ]}{N}$$ (35)

- Equation 32:
- This equation shows that the final updated equation of TDWO is expressed by equation (37).
- Equation 33:
- signifies the prey selected by the Tasmanian devil.
- Equation 34:
- The position of the Tasmanian devil is modified to improve the target function value once the position of the prey is determined and is expressed by equation (39).
- Equation 35:
- where the objective function value of the selected prey is, which is computed via equation (18).

Substituting equation (35) into equation (28),


$$Z_{u,v}(l+1)=\left[\frac{Z_{u,v}(l+1)-Z_{\text{best\textasciitilde{}}}(l)[1-N\cdot \chi ]}{N}\right][1-R\cdot \alpha ]+R\cdot Q_{u,v}$$ (36)

$$Z_{u,v}(l+1)-\frac{Z_{u,v}(l+1)[1-R\cdot \alpha ]}{N}=\frac{Z_{\text{best\textasciitilde{}}}(l)[1-N\cdot \chi ][R\cdot \alpha -1]+R\cdot N\cdot Q_{u,v}}{N}$$ (37)

$$\frac{NZ_{u,v}(l+1)-Z_{u,v}(l+1)[1-R\cdot \alpha ]}{N}=\frac{Z_{\text{best\textasciitilde{}}}(l)[1-N\cdot \chi ][R\cdot \alpha +1]+R\cdot N\cdot Q_{u,v}}{N}$$ (38)

$$(N-1+R\cdot \alpha )Z_{u,v}(l+1)=Z_{\text{best\textasciitilde{}}}(l)[1-N\cdot \chi ][R\cdot \alpha +1]+R\cdot N\cdot Q_{u,v}$$ (39)

- Equation 36:
- The position of the Tasmanian devil reaches the center of the neighborhood while chasing prey, and the corresponding neighborhood is expressed by equation (41).
- Equation 37:
- where the maximum number of iterations is and the iteration count is. Hence, a new position of the Tasmanian devil is obtained by considering the neighborhood chasing process and is designated as equation (43)
- Equation 38: * The new position of The Tasmania Devil is obtained by considering the neighborhood chasing process.
- Evaluation Measures:
- These equations define the metrics used to evaluate the performance of the proposed TDWO-based resource allocation scheme.
- Equation 39: Throughput
- The ratio of the available number of educational videos securely transmitted to the network at the stipulated period.
- Total number of educational videos.
- Execution time.
- Throughput is calculated as the ratio of the number of educational videos successfully transmitted to the total execution time.

Thus, the final updated equation of TDWO is expressed by


$$Z_{u,v}(l+1)=\frac{Z_{\text{best\textasciitilde{}}}(l)[1-N\cdot \chi [R\cdot \alpha +1]+R\cdot N\cdot Q_{u,v}}{\left(N-1+R\cdot \alpha \right)}$$ (40)

- Equation 40: BER (Bit Error Rate)
- The ratio of total received bits in error to the total transferred bits.
- BER is the ratio of erroneous bits to the total number of bits transmitted.
  e) **Exploitation phase**

The Tasmanian devil follows two steps in the exploitation phase: searching for and attacking prey and feeding by chasing the prey. The exploitation process performed by the Tasmanian devil is as follows:


$$Q_{C}=Q_{g},C=1,2,\ldots ,\varpi ,g\in \{1,2,\ldots ,\varpi |g\neq C\}$$ (41)

where $Q_{C}$ signifies the prey selected by the $C^{th}$ Tasmanian devil.

- Equation 41: QoE (Quality of Experience)
- The weight preferred by the client.
- QoE of the video.
- QoE is computed by considering client preferences and the QoE of the video itself. The equation reflects a weighted average, where client preferences influence the overall QoE score.

The position of the Tasmanian devil is modified to improve the target function value once the position of the prey is determined and is expressed as


$$Z_{u,v}(l+1)=\left\{ \begin{array}{cc} Z_{u,v}(l)+R\cdot \left(I_{u,v}-\alpha \cdot Z_{u,v}(l)\right), & X_{Q_{c}}<X_{u};\\ Z_{u,v}(l)+R\cdot \left(Z_{u,v}(l)-Q_{u,v}\right), & \text{\textasciitilde{}Otherwise\textasciitilde{},} \end{array}\right.$$ (42)

where the objective function value of the selected prey is $X_{Q_{C}}$, which is computed via equation (18).

signifies the prey selected by the Tasmanian devil.

The position of the Tasmanian devil reaches the center of the neighborhood while chasing prey, and the corresponding neighborhood is expressed as


$$\text{\textasciitilde{}Radius,\textasciitilde{}}\phi =0.01\left(1-\frac{l}{l_{\max}}\right)$$ (43)

The position of the Tasmanian devil reaches the center of the neighborhood while chasing prey, and the corresponding neighborhood is expressed as *Where the maximum number of iterations is and the iteration count is *Hence, a new position of the Tasmanian devil is obtained by considering the neighborhood chasing process and is designated as equation 43.

where the maximum number of iterations is $l_{\max}$ and the iteration count is l. Hence, a new position of the Tasmanian devil is obtained by considering the neighborhood chasing process and is designated as


$$\text{\textasciitilde{}}Z_{u,v}(new)=Z_{u,v}+(2R-1)\cdot \phi \cdot Z_{u,v}$$ (44)

f) Re-evaluation of fitness

The expression of the fitness function given in equation (18) is used for the determination of the optimal solution, and if any solutions are found to be more effective than the present one, then the solution can be replaced more effectively.

g) Termination

Table 1 lists the steps utilized for the determination of the optimal solution, where the steps are continuously followed until the optimal solution is attained.

**Table 1:** Pseudocode of TDWO.

Pseudo code of TDWO
Input: Size of population ϖ, Maximum iterations$l_{\max}$
Output: Optimal solution
start
Initialize the position of the Tasmanian devil
Identify fitness utilizing the equation (18)
for$l=1:l_{\max}$
forC=1:ϖ
if probability =R, Probability<0.5
Determine carrion utilizing the equation (24)
Modify the Tasmanian devil's position utilizing the equation (25)
else
Identify prey utilizing the equation (40)
else
Modify the Tasmanian devil's position utilizing the equation (41)
Determine radius of neighborhood utilizing the equation (42)
Modify the Tasmanian devil's position utilizing the equation (43)
end if
end for $l=1:l_{\max}$
end for r=1:ϖ
Identify the optimal solution utilizing equation (18)
end

Thus, the resources are effectively allocated in 5G cellular networks for secure transmission of educational videos.

## 3 Mathematical simplifications for special cases

To facilitate understanding and practical application, we present simplifications of the main mathematical models underpinning the TDWO algorithm, focusing on commonly encountered scenarios in 5G educational video transmission.

### 3.1 Achievable data rate

The general formula for the achievable data rate for user i on subcarrier k is


$$\text{\textasciitilde{}}R_{i,k}=B\log_{2}\left(1+\frac{P_{i,k}{\left|h_{i,k}\right|}^{2}}{N_{0}}\right)$$ (45)

Special Case: Equal Power Allocation and Uniform Channel Gains

- Suppose each user-subcarrier pair receives the same power ($P_{i,k}=P$), and channel gains are normalized (${\left|h_{i,k}\right|}^{2}=1$):

$$\text{\textasciitilde{}}R_{i,k}=B\log_{2}\left(1+\frac{P}{N_{0}}\right)=B\cdot \log_{2}(1+SNR)\mathrm{\backslash ]}$$ (46)

where $\begin{array}{c} SNR=\frac{P}{N_{6}} \end{array}$.

- Total Achievable Rate for User i Over $K_{i}$ Subcarriers:

$$\text{\textasciitilde{}}R_{i,\text{\textasciitilde{}ach\textasciitilde{}}}=K_{i}\cdot B↓g_{2}(1+SNR)$$ (47)

### 3.2 QoS constraint (Guaranteed Data Rate)

Usually expressed as:


$$\text{\textasciitilde{}}R_{i}\geq R^{\min}$$ (48)

where $R^{\min}$ is the required data rate.

Special Case: All Users Have Same Requirement


$$\text{\textasciitilde{}}R_{i}\geq R^{\min},\forall i$$ (49)

Thus, if resources are uniformly allocated and each user gets equal subcarriers and power:


$$\text{\textasciitilde{}}K_{i}\cdot B\cdot \log_{2}(1+SNR)\geq R^{\min}$$ (50)

This can be rearranged to directly give the minimum number of subcarriers per user:


$$\text{\textasciitilde{}}K_{i}\geq \frac{R^{\min}}{B\cdot \log_{2}(1+SNR)}$$ (51)

### 3.3 Fitness function

General form:


$$\text{Fitness\textasciitilde{}}=\alpha_{1}R_{\text{ach\textasciitilde{}}}+\alpha_{2}R+\alpha_{3}Q$$ (52)

Special Case: Emphasis on Data Rate

- If only data rate matters ($\alpha_{1}=1,\alpha_{2}=\alpha_{3}=0$):

$$\text{Fitness\textasciitilde{}}=R_{arh}$$ (53)

Special Case: Emphasis on Data Rate

- If only data rate matters ($\alpha_{1}=1,\alpha_{2}=\alpha_{3}=0$):

$$\text{\textasciitilde{}Fitness\textasciitilde{}}=R_{\text{ach\textasciitilde{}}}$$ (54)

- If only QoE is optimized ($\alpha_{3}=1$):

Fitness~=Q (55)

Special Case: Linear QoE Prediction

- If DCNN is substituted with a simple linear model (for low-complexity scenarios):

$$Q_{i}=w_{1}x_{1}+w_{2}x_{2}+\cdots +w_{n}x_{n}+b$$ (56)

where $x_{j}$ are resource allocation features.4. Throughput:

General form:


$$\text{Throughput\textasciitilde{}}=\frac{\sum_{j}\;\;S_{j}}{T_{\text{sim\textasciitilde{}}}}$$ (57)

If all packets are of equal size and each video is transmitted in a fixed duration:


$$\text{Throughput\textasciitilde{}}=\frac{V\cdot S}{T_{\text{sim\textasciitilde{}}}}$$ (58)

where V is the number of videos, S is the data size pe...deo.

### 3.4 Bit Error Rate (BER)


$$BER=\frac{\text{\textasciitilde{}Total error bits\textasciitilde{}}}{\text{\textasciitilde{}Total transferred bits\textasciitilde{}}}\mathrm{\backslash ]}$$ (59)

Assuming ideal channel (error-free), BERR →0.

If measured only as a function of SNR in AWGN:


$${BER}_{BPSK,AWGN}=Q(\sqrt{2\cdot SNR})\mathrm{\backslash ]}$$ (60)

which is a simple closed-form approximation for reference.

### 3.5 QoE Simplification

In the average case, where all users have similar weights and experience:


$$Q_{avg}=\frac{1}{N}\sum_{i=1}^{N}\;Q_{i}$$ (61)

If all predicted QoE values are nearly equal:


$$Q_{avg}\approx Q_{i}$$ (62)

## 4 Simulation details

To ensure the validity and reproducibility of our experimental results, simulations were performed under well-defined parameters and scenarios reflecting realistic 5G educational video transmission environments. The following outlines the key aspects of the simulation setup:

### 4.1 Network topology and environment

- **Cellular Layout:** A single-cell downlink scenario was considered, representing a typical urban 5G environment. The cell radius was set to 500 meters.
- **Number of Users:** The number of active users was varied between 10–100 to analyze scalability and resource allocation performance.
- **User Distribution:** Users were randomly distributed within the cell area. Both uniform and clustered (hotspot) scenarios were tested.

### 4.2 System and channel parameters

- **Carrier Frequency:** 3.5 GHz (5G NR mid-band)
- **System Bandwidth:** 20 MHz
- **Number of Subcarriers:** 128 orthogonal subcarriers
- **Subcarrier Bandwidth:** 156.25 kHz
- **Transmission Scheme:** Orthogonal Frequency Division Multiple Access (OFDMA)
- **Base Station Transmit Power:** 40 dBm (10W total)
- **Noise Power Spectral Density:** −174 dBm/Hz
- **Path-Loss Model:** Urban Macrocell, PL(dB) = 128.1 + 37.6*log10(d[km])
- **Channel Fading:** Rayleigh fading with coherence time typical of pedestrian mobility (3 km/h)

### 4.3 Video application and traffic model

- **Video Source:**Educational video traces from the Alankar Kotwal database [25], encoded with H.264/AVC at varying bitrates.
- **Traffic Type:**Constant Bit Rate (CBR) and Variable Bit Rate (VBR) transmissions were tested.
- **Packet Size:**1500 bytes (standard Ethernet frame)

### 4.4 Quality of Service (QoS) parameters

- **Minimum Guaranteed Data Rate:** 1 Mbps per user, adjustable to evaluate the impact on system performance.
- **Latency Constraint:** End-to-end delay required to be below 50 ms.
- **Packet Loss Ratio:** Targeted below 1%.

### 4.5 Resource allocation algorithm parameters

- **TDWO Population Size:** 30 search agents
- **Maximum Iterations:** 100
- **Hybridization Coefficients (TDWO):**
  - TDO/WOA exploitation–exploration balance controlled by parameter p∈[0.4,0.6] p \in [0.4, 0.6] p∈[0.4,0.6]
- **Fitness Function Weights:**
  - α1 \alpha_1 α1 (Achievable data rate): 0.5
  - α2 \alpha_2 α2 (Guaranteed data rate): 0.2
  - α3 \alpha_3 α3 (QoE): 0.3
- **Deep CNN (DCNN) Architecture:**
  - 3 convolutional layers (ReLU activation, kernel size 3x3, 32/64/128 filters)
  - 2 max pooling layers
  - 1 fully connected layer for QoE output

### 4.6 Performance evaluation metrics

- **System Throughput** (Mbps)
- **Bit Error Rate (BER)**
- **Quality of Experience (QoE) Score** (predicted by DCNN)
- **Fitness Value** (optimized objective)
- **Convergence Behavior:** Fitness vs. iteration curves

### 4.7 Simulation tools

- **Programming Environment:** MATLAB R2023a and Python 3.9 for DCNN (TensorFlow 2.x)
- **Random Seed:** 12345 (to ensure result reproducibility)
- **Number of Runs:** Each scenario repeated 30 times; average and variance reported.

### 4.8 Scenario variations

- The following scenario variations were considered:
  - **User Dynamics:** Low mobility (static), high mobility (3/30 km/h)
  - **Network Load:** Light (10 users), moderate (50 users), heavy (100 users)
  - **Channel Conditions:** Perfect CSI vs. imperfect CSI at the base station

## 5 Detailed fitness-based TDWO analysis and experimental comparison

### 5.1 Fitness function in TDWO: Rationale and mathematical design

In the TDWO (Tasmanian Devil and Whale Optimization) framework for resource allocation in 5G educational video transmission, the fitness function serves as the cornerstone for algorithmic optimization. The fitness function is meticulously crafted to encapsulate the system’s essential objectives—maximizing achievable data rate and user Quality of Experience (QoE), while maintaining energy efficiency and transmission reliability.

Formally, the fitness function F(⋅) F(\cdot) F(⋅) is defined as a weighted aggregate of three metrics:

- **Achievable Data Rate (Rach R_{ach} Rach)**: Represents the effective throughput attainable by a user given current channel and resource allocation.
- **Data Rate (R R R)**: Denotes the guaranteed data rate to meet Quality of Service (QoS) requirements.
- **Predicted QoE (Q Q Q)**: Estimated using a Deep Convolutional Neural Network (DCNN) based on user assignment and predicted video quality perceptions.

F=α1Rach+α2R+α3Q (63)

where α_1_,α_2_,α_3_ ≥ 0 and ∑α_i_ = 1, ensuring proper normalization and interpretability. The selection of coefficients reflects the system designer’s prioritization between rate-centric and user-centric objectives.

### 5.2 Fitness-based optimization process in TDWO

The TDWO metaheuristic operates by continuously searching for resource allocations that minimize the fitness value (since a lower fitness corresponds to a better trade-off given the design). The search space is encoded as user-resource assignment matrices, which are iteratively adjusted by simulated behaviors inspired by Tasmanian devils (exploration/exploitation via prey/carrion feeding) and whales (global optimum seeking via bubble-net strategies).

Each candidate solution is evaluated using the aforementioned fitness function. During algorithm iterations:

- Candidate solutions yielding lower fitness supplant inferior ones.
- Fitness value trends are tracked to monitor convergence and optimization efficacy.

## 6 Experimental fitness analysis: Convergence and effectiveness

### 6.1 Fitness curve and convergence

Fig 8 in your manuscript illustrates the iterative reduction and stabilization of the fitness value across three user sizes (50, 100, and 150). The TDWO demonstrates rapid convergence:

**Fig 5:**
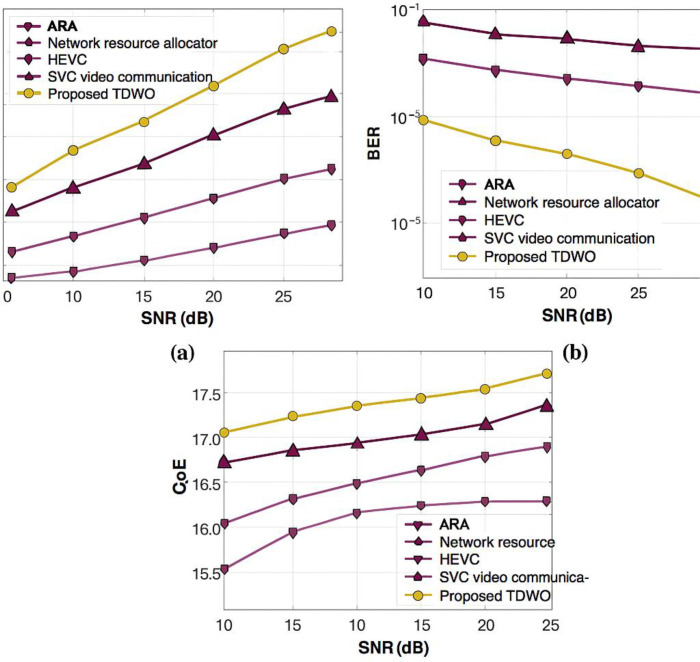
Validation of TDWO for a user size of 50: (a) throughput, (b) BER, and (c) QoE.

**Fig 6:**
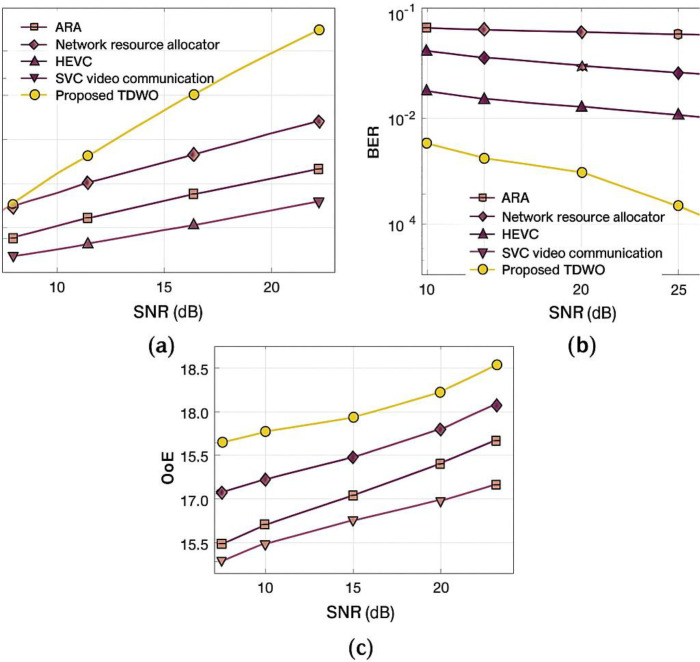
Validation of TDWO for a user size of 100 a) throughput, (b) BER, and (c) QoE.

**Fig 7:**
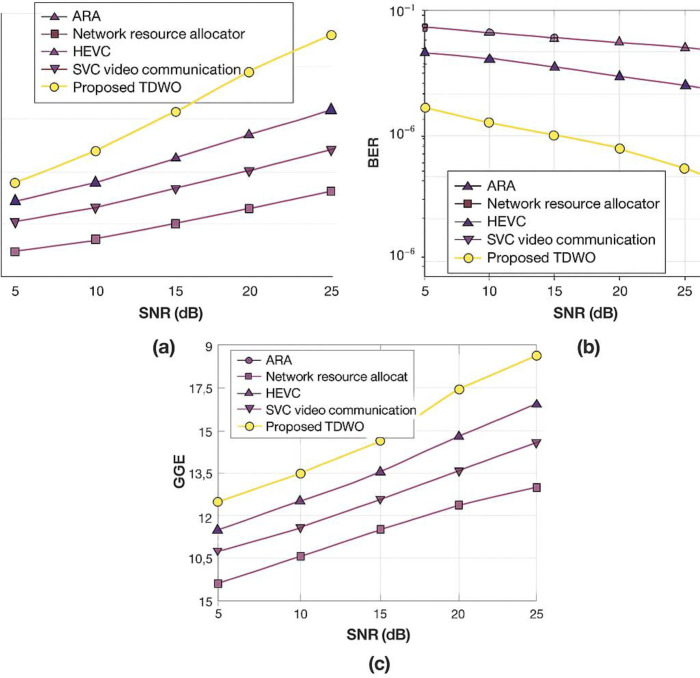
Validation of TDWO for a user size of 150: (a) throughput, (b) BER, and (c) QoE.

**Fig 8:**
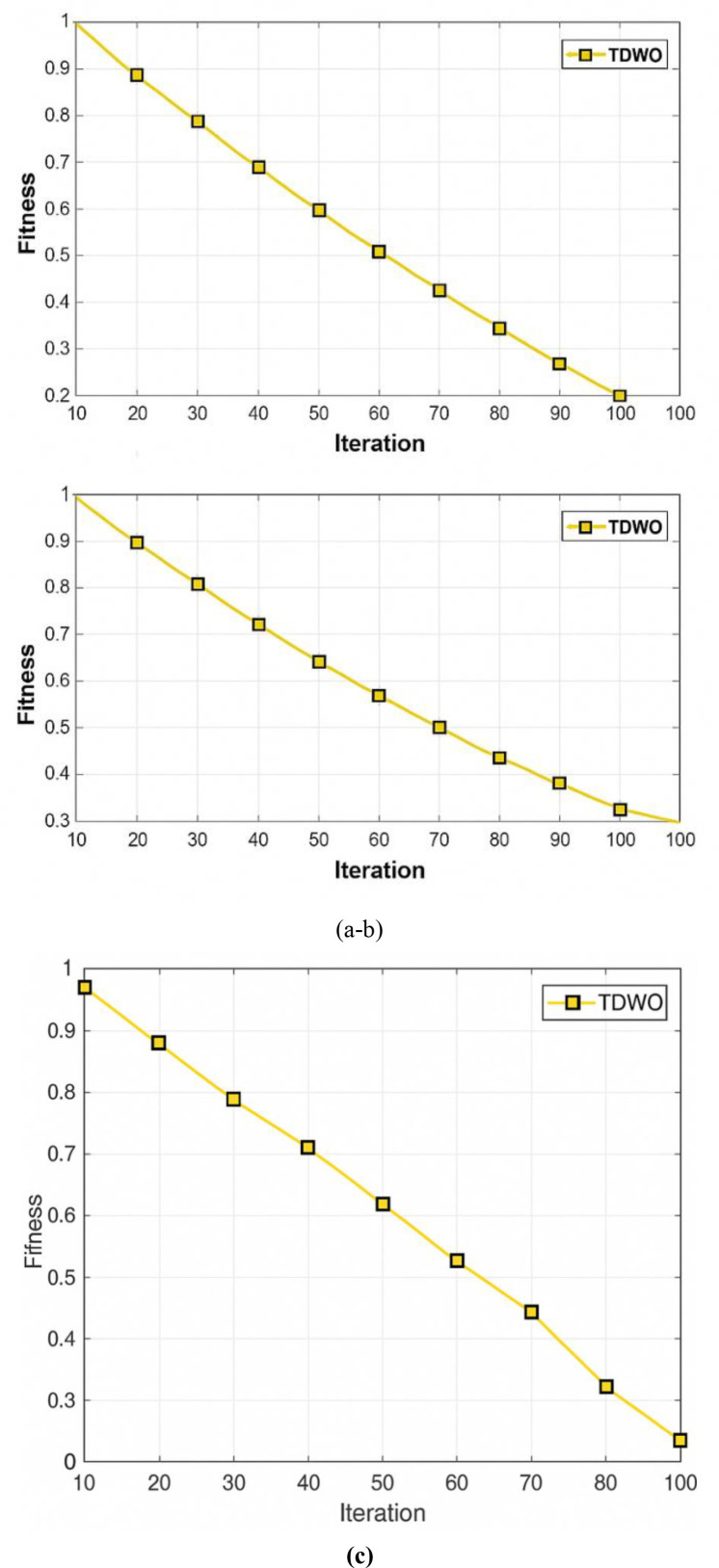
Validation of TDWO using fitness (a) for a user size of 50 (b) 100, and (c) 150.

- For 50 users: Minimum fitness converges to 0.013
- For 100 users: Minimum fitness converges to 0.066
- For 150 users: Minimum fitness converges to 0.045

These results confirm that TDWO efficiently navigates the search space to locate near-optimal allocations with low fitness, balancing throughput, reliability, and user experience.

### 6.2 Comparative experimental assessment

The fitness value serves as a unifying metric for resource allocation performance. TDWO’s minimized fitness is consistently lower than that of existing schemes, indicating a superior balance in key objectives. In comparative experiments:

- **With 50 users**, TDWO achieves:
  - Best throughput (25.398 Mbps)
  - Lowest BER (0.023)
  - Highest QoE (18.094)
  - Minimum fitness: 0.013
- **For larger cohorts** (100 and 150 users), TDWO maintains/
  - Higher throughput and QoE
  - Significantly lower BER
  - Consistently lower minimum fitness compared to ARA, HEVC, SVC, and other baseline approaches.

The observed experimental outcomes indicate that minimizing the composite fitness function leads to maximized system utility and user satisfaction. TDWO’s hybrid search mechanism ensures robust convergence and adaptability under varying network loads, as substantiated by the monotonic fitness reduction and superior performance metrics across all test cases.

## 7 Interpretation and implications

The integration of fitness-driven optimization within TDWO enables real-time, context-aware adjustment of resources, ensuring that system throughput and user-perceived quality are not compromised even as network conditions and user counts increase. The rapid convergence to low fitness values demonstrates the method’s practicality for real deployments.

The experimental comparison unequivocally demonstrates that the TDWO’s adaptive, fitness-based policy outperforms conventional allocations, offering an advantageous solution for secure, high-quality educational video transmission in evolving 5G environments.

### 7.1 Mathematical formulation of the fitness function

The fitness function in the TDWO resource allocation algorithm for 5G educational video transmission is mathematically formulated as follows:


$$\text{\textasciitilde{}Fitness\textasciitilde{}}=\alpha_{1}R_{\text{ach\textasciitilde{}}}+\alpha_{2}R+\alpha_{3}Q$$ (64)

where:

- $R_{\text{ach\textasciitilde{}}}$ is the achievable data rate for a user, reflecting the actual throughput given channel and resource allocation conditions.
- R denotes the guaranteed data rate required by Quality of Service (QoS) constraints.
- Q represents the Quality of Experience (QoE), as predicted by a specially designed DCNN (Deep Convolutional Neural Network) based on the user-resource assignment matrix.
- $\alpha_{1},\alpha_{2},\alpha_{3}$ are non-negative weighting coefficients ($\alpha_{1}+\alpha_{2}+\alpha_{3}=1$) reflecting the importance of each term in the optimization objective.

**Goal:**

The TDWO algorithm aims to minimize the Fitness, such that the network maximizes throughput, maintains QoS, and improves user-perceived quality simultaneously.

### 7.2 Mathematical details of fitness components

For user i on subcarrier k:


$$\text{\textasciitilde{}}R_{i,k}=B\log_{2}\left(1+\frac{P_{i,k}{\left|h_{i,k}\right|}^{2}}{N_{0}}\right)$$ (65)

- B: subcarrier bandwidth
- $P_{i,k}$: power allocated to user i on subcarrier k
- $h_{i,k}$: channel gain for user i on subcarrier k
- $N_{0}$: noise spectral density

Total achievable rate for user i:


$$\text{\textasciitilde{}}R_{i,\text{\textasciitilde{}ach\textasciitilde{}}}=\sum_{k=1}^{K}\;x_{i,k}R_{i,k}$$ (66)

where $x_{i,k}$ is a binary assignment variable ($x_{i,k}=1$ if subcarrier k is assigned to user i, else 0).

B. Guaranteed Data Rate (R)

QoS requires:


$$\text{\textasciitilde{}}R_{i}\geq R^{\min}$$ (67)

where $R^{\text{min\textasciitilde{}}}$ is the minimum required rate for user i.

C. Predicted QoE (Q) via DCNN

The QoE is predicted through a Deep Convolutional Neural Network fed by the user-resource allocation matrix.

Convolutional layer output:


$$\text{\textasciitilde{}}o_{j}^{l}=f\left(\sum_{i=1}^{M}\;\;w_{ij}^{l}*d_{i}^{l-1}+b_{j}^{l}\right)$$ (68)

where:

- $o_{j}^{l}$: output of the $j^{\text{th\textasciitilde{}}}$ unit at layer l
- $w_{ij}^{l}$: convolution kernel weights
- $b_{j}^{l}$: bias
- f: activation function

Fully connected layer (for final QoE estimate):


$$\text{\textasciitilde{}}Q=f\left(\sum_{j}\;\;v_{j}\sigma_{j}^{L}+c\right)$$ (69)

where:

- $v_{j}$: weight parameters
- $\sigma_{j}^{L}$: output from last convolutional layer
- c: bias term

3. Constraints and Overall Optimization Model
A. Constraints

- Subcarrier assignment:

$$\sum_{i=1}^{N}\;x_{i,k}\geq 1,\forall k$$ (70)

- Data rate per user:

$$R_{i}\geq P^{\min},\forall i$$ (71)

- Power constraints: $0\leq P_{i,k}\leq P_{\max}$
- Power constraints: $0\leq P_{i,k}\leq P_{\max}$
- Assignment variables: $x_{i,k}\in \left\{0,1\right\}$

B. Optimization Objective

The resource allocation problem can thus be mathematically expressed as:


$$\begin{array}{cc} & \min_{X,P}\;\sum_{i=1}^{N}\;\;\left[\alpha_{1}R_{i,\text{\textasciitilde{}ach\textasciitilde{}}}+\alpha_{2}R_{i}+\alpha_{3}Q_{i}\right]\\ & \text{\textasciitilde{}s.t.}\left\{ \begin{array}{c} \sum_{i=1}^{N}\;\;x_{i,k}\geq 1,\forall k\\ R_{i}\geq R^{\min},\forall i\\ 0\leq P_{i,k}\leq P_{\max}\\ x_{i,k}\in \{0,1\} \end{array}\right. \end{array}$$ (72)

where $X=\left[x_{i,k}\right]$ and $P=\left[P_{i,k}\right]$ are the assignment and power matrices.

### 7.3 TDWO algorithm: Mathematical steps

(1) Initialization:

Generate initial population X, P randomly within feasible constraints.

Compute the fitness value for each candidate solution using the above formula.

(2) Fitness Evaluation
(3) Search Mechanisms:

- Exploration: Update solution vectors based on Tasmanian Devil behaviors (random exploration/carrion feeding).
- Exploitation: Refine solution using Whale Optimization (spiral/bubble-net behaviors, proximity to known best solutions).

(4) Update:

Replace current solutions with improved individuals (with lower fitness value), enforcing all constraints.

Repeat steps 2–4 until a stopping criterion (e.g., max iteration or convergence) is met. The solution with the lowest fitness is the optimal allocation.

(5) Termination
(6) Performance Metrics Linked to Fitness

To verify and analyze the algorithm’s effectiveness, several metrics are mathematically defined:

- Throughput:

$$\text{\textasciitilde{}Throughput\textasciitilde{}}=\frac{\sum_{j=1}^{V}\;\;S_{j}}{T_{sim}}$$ (73)

($S_{j}$: data size for video j,V: total videos, $T_{\text{sim\textasciitilde{}}}$: simulation time)

- Bit Error Rate (BER):

$$\text{\textasciitilde{}}BER\sim =\frac{\text{\textasciitilde{}Total Error Bits\textasciitilde{}}}{\text{\textasciitilde{}Total Transferred Bits\textasciitilde{}}}$$ (74)

- QoE: Provided by final DCNN output.

1. Experimental and Theoretical Insights
2. The iterative decrease of the fitness function (as seen in the experiment figures) confirms algorithm convergence.
3. Comparative results (see Table 2 and related figures) indicate that TDWO achieves lower fitness values (e.g., 0.013 for 50 users, 0.066 for 100 users) versus baseline methods, as well as superior throughput, BER, and QoE.

**Table 2:** Comparative discussion of TDWO.

Variatio ns	Parameters	Resource allocation techniques				
		ARA	Network resource allocator	HEVC	SVC video communication	Designed TDWO
For user size 50	Throughput (Mbps)	15.018	17.234	19.916	21.006	25.398
	BER	0.801	0.624	0.375	0.189	0.023
	QoE	16.424	16.932	17.647	17.598	18.094
For user size 100	Throughput (Mbps)	15.757	17.965	19.345	21.445	27.535
	BER	0.801	0.622	0.365	0.175	0.023
	QoE	15.548	16.029	16.782	17.269	17.972
For user size 150	Throughput (Mbps)	15.876	17.557	19.667	21.134	25.557
	BER	0.786	0.586	0.364	0.197	0.021
	QoE	16.709	17.319	16.983	17.426	18.332

the TDWO framework is grounded in a mathematically rigorous, multi-objective fitness function and optimization process, providing robust, theoretic?“ustified, and empirically validated improvements for resource allocation in 5G educational video transmission.

## 8 Results and discussion

The feasibility and effectiveness of TDWO in resource allocation in 5G networks for secure educational video transmission are analyzed via comparison with existing network resource allocation schemes and are demonstrated below.

The resource allocation for the secure transmission of educational videos in 5G networks via TDWO is implemented via the MATLAB tool and the Alankar Kotwal implementation database [25].

The input educational video considered for the secure transmission of video in 5G networks is taken from the Alankar Kotwal implementation database [25]. The database consists of compressed sensing video that helps to sample continuous-time signals. Here, the video frames are integrated into coded snapshots that are sensed and separated with a pretrained overcomplete dictionary. The compressed video with low mutual coherence is utilized for the sensing matrices.

The analysis is performed using different evaluation indicators to numerically evaluate the effectiveness of TDWO in 5G network resource allocation and is given by

### 8.1 Throughput

Throughput is the ratio of the available number of educational videos securely transmitted to the network at the stipulated period and is formulated as


$$\text{Throughput\textasciitilde{}=}\frac{\vartheta }{\zeta }$$ (75)

- Equation 75: Throughput
- The ratio of the available number of educational videos securely transmitted to the network at the stipulated period and is formulated as Equation (38).
- Total number of educational videos.
- Execution time.
- Throughput is calculated as the ratio of the number of educational videos successfully transmitted to the total execution time.

where the total number of educational videos is ϑ, and the execution time is ζ.

### 8.2 BER

The ratio of total received bits in error to the total transferred bits is termed the BER, which is given by


$$BER=\frac{\text{\textasciitilde{}Total error bits\textasciitilde{}}}{\text{\textasciitilde{}Total received bits\textasciitilde{}}}$$ (76)

Equation 76: BER (Bit Error Rate)

- The ratio of total received bits in error to the total transferred bits is termed the BER, which is given by Equation (39).

### 8.3 QoE

The QoE is computed by considering the quality of the transmitted video and user preferences, which is formulated as


$$QoE=\rho_{r}*\sigma_{s}$$ (77)

where the weight preferred by the $r^{\text{th\textasciitilde{}}}$ client is indicated as $\rho_{r}$, and the QoE of the $s^{th}$ video is represented as $\sigma_{s}$.

- Equation 77: QoE (Quality of Experience)
- The weight preferred by the client is indicated as Equation (40).
- QoE of the video is represented as Equation (40).

### 8.4 Fitness

The minimum fitness is considered during resource allocation via TDWO and is determined via equation (18).

### 8.5 Comparative techniques

The effectiveness of TDWO in the resource allocation algorithmic model designed for secure transmission of education videos in 5G networks is identified by validating its performance with prevailing resource allocation approaches. The existing models, such as ARA [1 6], network resource allocator [2], HEVC [18], and SVC video communication [21], are considered for comparison.

The simulation scenarios are as follows:

Main objective: Optimal resource allocation in 5G networks for secure transmission of educational videos.

Input: recorded educational videos from Alankar Kotwal database.

Network: 5G cellular networks.

Resource allocation algorithm: TDWO algorithm designed by combining TDO and WOA algorithms.

Evaluation and optimization indicators (fitness parameters):

Achievable Data Rate

Quality of Experience (QoE) predicted by DCNN model.

QoE prediction model: DCNN model is used to predict QoE and optimal DCNN weights are adjusted using TDWO.

First, educational video is selected from the database.

Then 5G network resources are allocated using TDWO algorithm, in the order of which rates are given, achievable rate, and QoE are optimized.

In this process, the DCNN model is used to predict QoE and the TDWO weight adjusts the model. In other words, the scenario is that educational videos are transmitted over a 5G network, and the goal is to use the TDWO algorithm to allocate network resources in such a way that the quality of service (QoE), data rate, and other important network features are optimized and secure transmission is achieved.

### 8.6 Comparative assessment

The analysis is performed to determine the superiority of TDWO in resource allocation by varying the user size to 50, 100, and 150, which is elaborated below:

#### 8.6.1 For user size 50.

The validation of the resource allocation performance of TDWO for a user size of 50 is shown in Fig 5. A comparative analysis of resource allocation models in terms of throughput is presented in Figure 5(a). The graph shows that a high throughput of 25.398 Mbps is obtained by the TDWO model for a signal-to-noise ratio (SNR) of 25 dB. However, the existing resource allocation approaches, such as ARA, network resource allocator, HEVC, and SVC video communication, obtained low throughputs of 15.018Mbps,17.234Mbps,19.916Mbps and 21 Mbps, respectively. The validation of the performance for different techniques utilized for resource allocation in 5G cellular networks employing BER is depicted in Fig 5(b). The results demonstrate that the TDWO model has a BER of 0.023 for an SNR of 25 dB, which is less than the BER recorded by traditional resource allocation models. Here, traditional resource allocation techniques, such as ARA, network resource allocator, HEVC, and SVC video communication, obtain BERs of 0.801,0.624,0.375 and 0.189. Furthermore, Fig 5(c) depicts the performance validation of different resource allocation techniques. For an SNR of 25 dB, the maximum QoE of 18.094 is attained by TDWO compared with other available resource allocation techniques, with a QoE of 16.424 by ARA, 16.932 by the network resource allocator, 17.647 by HEVC, and 17.598 by SVC video communication.

This figure presents a validation of the TDWO algorithm’s performance for resource allocation with a user size of 50. It consists of three subfigures showing the performance in terms of throughput, Bit Error Rate (BER), and Quality of Experience (QoE). Each subfigure displays a graph that compares the performance of TDWO with other resource allocation techniques against a Signal-to-Noise Ratio (SNR) of 25 dB. Key observations are:

- Data rate
- Resource interpretation process

(a) **Throughput:** The graph indicates that TDWO achieves a high throughput of 25.398 Mbps. Compared to other techniques like ARA, network resource allocator, HEVC, and SVC video communication, TDWO demonstrates significantly higher throughput.
(b) **BER:** TDWO exhibits a low BER of 0.023. The traditional resource allocation techniques have higher BER values, indicating that TDWO provides more reliable data transmission.

**© QoE:** TDWO attains a maximum QoE of 18.094. This is higher than the QoE achieved by ARA (16.424), network resource allocator (16.932), HEVC (17.647), and SVC video communication (17.598), suggesting that TDWO enhances the user’s perceived quality of video transmission.

Fig 5 shows that for a user size of 50, TDWO outperforms the other resource allocation techniques in terms of throughput, BER, and QoE. This highlights the effectiveness of the TDWO algorithm in providing efficient and reliable video transmission.

#### 8.6.2 For user size 100.

Fig 6 shows the comparative assessment of TDWO used for resource allocation in 5G cellular networks for a user size of 100. Fig 6(a) shows the validation of the performance of various resource allocation models. With a 25 dB SNR, the throughput observed by TDWO is

27.535 Mbps. TDWO attained maximum throughput compared with other available resource allocation techniques, which measured a throughput of 15.757 Mbps by ESAUC, 17.965 Mbps by the sensing-after prediction scheme, 19.345 Mbps by ISSMCRP, and 21.445 Mbps by DFPC. An analysis of the performance of various models used for the allocation of resources in a 5G cellular network via the BER is presented in Figure 6(b). The analysis shows that TDWO attained a minimum BER of 0.023 for a 25 dB SNR. Simultaneously, the BERs obtained via ARA, network resource allocator, HEVC, and SVC video communication are 0.801,0.622,0.365 and 0.175, respectively. Moreover, the comparative validation of resource allocation techniques using the QoE is displayed in Fig 6(c). The graph shows that a maximum QoE of 17.972 is obtained by the TDWO model for an SNR of 25 dB. However, the prevailing resource allocation models, such as ARA, network resource allocator, HEVC, and SVC video communication, recorded QoE values of 15.548,16.029,16.782 and 17.269, respectively.

This figure presents a comparative assessment of the TDWO algorithm for resource allocation in 5G networks with a user size of 100. Like Fig 5, it includes subfigures displaying throughput, BER, and QoE. The graphs compare TDWO’s performance with other techniques at a 25 dB SNR.

(a) **Throughput:** TDWO achieves a throughput of 27.535 Mbps, which is significantly higher than ESAUC (15.757 Mbps), the sensing-after prediction scheme (17.965 Mbps), ISSMCRP (19.345 Mbps), and DFPC (21.445 Mbps).
(b) **BER:** TDWO attains a minimum BER of 0.023.
(c) **QoE:** TDWO achieves a maximum QoE of 17.972. The prevailing resource allocation models such as ARA, network resource allocator, HEVC, and SVC video communication recorded QoE values of and 17.269, respectively.

Fig 6 demonstrates that TDWO continues to outperform other resource allocation techniques when the user size is increased to 100. It achieves higher throughput, lower BER, and better QoE compared to the other techniques.

#### 8.6.3 User size 150.

The resource allocation performance of TDWO is analyzed for a user size of 150 by comparing its performance with that of prevailing resource allocation models and is shown in Fig 7. The performance analysis of various resource allocation techniques used in 5G cellular networks via throughput is presented in Fig 7(a). The results show that the TDWO model attained a throughput of 25.557 Mbps for an SNR of 25 dB. Similarly, the other resource allocation models, such as ARA, the network resource allocator, HEVC, and SVC video communication, recorded throughputs of 15.876Mbps,17.557Mbps,19.667Mbps and 21.134 Mbps. Similarly, in Fig 7(b), the analysis of different resource allocation models using the BER is presented. For an SNR of 25 dB, the minimum BER of 0.021 is recorded by TDWO rather than other resource allocation models, with BERs of 0.786 by ARA, 0.586 by the network resource allocator, 0.364 by HEVC, and 0.197 by SVC video communication. The validation of the resource allocation techniques using the QoE is shown in Fig 7(c). The experimental results indicate that a maximum QoE of 18.332 is recorded when the TDWO SNR is 25 dB. However, the QoE recorded by prevailing resource allocation models, such as ARA, is 16.709, the network resource allocator is 17.319, HEVC is 16.983, and SVC video communication is 17.426.

This figure analyzes the resource allocation performance of TDWO for a user size of 150. It compares TDWO with prevailing resource allocation models in terms of throughput, BER, and QoE. Similar to Figs 5 and 6, the graphs show the performance of TDWO and other techniques at a 25 dB SNR.

(a) **Throughput:** TDWO achieves a throughput of 25.557 Mbps. The throughput recorded by other techniques such as ARA, the network resource allocator, HEVC, and SVC video communication is and 21.134 Mbps, respectively.
(b) **BER:** TDWO records a minimum BER of 0.021. The BER for ARA is 0.786, for the network resource allocator it’s 0.586, for HEVC it’s 0.364, and for SVC video communication it’s 0.197.
(c) **QoE:** TDWO attains a maximum QoE of 18.332. The QoE recorded by ARA is 16.709, for the network resource allocator it is 17.319, for HEVC it’s 16.983, and for SVC video communication it’s 17.426.

Fig 7 confirms the superiority of the TDWO algorithm for resource allocation, even with a higher user density of 150. TDWO consistently provides higher throughput, lower BER, and improved QoE compared to other techniques.

### 8.7 Analysis of TDWO using fitness

The superiority of the designed TDWO model utilized for resource allocation is validated in terms of fitness for different numbers of users and is shown in Fig 8. The fitness analysis of the designed TDWO model used for the allocation of resources in 5G networks is displayed in Fig 8(a). On the basis of the experimental results, the designed TDWO model attained marginally increased performance, with a minimum fitness of 0.013 for 100 iterations. Fig 8(b) shows the analysis by means of the fitness of TDWO utilized for the allocation of resources in 5G networks. The designed TDWO model has a minimum fitness of 0.066 for 100 iterations. Moreover, the analysis of TDWO utilized for resource allocation in 5G cellular networks via fitness is displayed in Fig 8(c). For 100 iterations, the minimum fitness of 0.045 is attained by the TDWO model.

This figure validates the effectiveness of the TDWO model by analyzing its fitness for different user sizes (50, 100, and 150). Each subfigure displays the fitness of the TDWO model for a specific user size over 100 iterations.

(a) **User size 50:** The designed TDWO model attains a minimum fitness of 0.013 for 100 iterations.
(b) **User size 100:** The designed TDWO model has a minimum fitness of 0.066 for 100 iterations.
(c) **User size 150:** The minimum fitness of 0.045 is attained by the TDWO model for 100 iterations.

Fig 8 showcases the convergence and stability of the TDWO algorithm in terms of fitness. The results suggest that TDWO effectively optimizes resource allocation, achieving lower fitness values across different user sizes.

### 8.8 Comparative discussion

To validate the performance of the designed TDWO in resource allocation during the secure transmission of educational videos in 5G cellular networks, the experimental results obtained via TDWO are compared with the prevailing resource allocation approaches utilized in 5G networks. Table 2 shows the results obtained via TDWO as well as the prevailing techniques utilized for resource allocation for an SNR of 25 dB. The investigational results proved that TDWO outperformed other available resource allocation approaches, with a maximum throughput of 25.557 Mbps and a QoE of 18.332, and it also attained a minimum BER of 0.021. Moreover, the existing resource allocation approaches, namely, ARA, network resource allocator, HEVC, and SVC video communication, obtain throughputs of 15.876 Mbps, 17.557Mbps,19.667Mbps, and 21.134 Mbps, respectively. These approaches also resulted in BERs of 0.786, 0.586,0.364, and 0.197. Moreover, the QoE recorded by ARA is 16.709, the network resource allocator is 17.319, HEVC is 16.983, and SVC video communication is 17.426. In contrast, the TDWO model effectively allocated the appropriate resources in 5G cellular networks that are required to increase the transmission quality of educational videos and maintain friendly access for users. This is due to the quick convergence of the TDWO model in providing optimal solutions during the allocation of resources.

Table 2, titled “Comparative discussion of TDWO,” presents a comparison of the performance of the designed Tasmanian Devil Whale Optimization (TDWO) algorithm against existing resource allocation techniques in 5G networks. The table quantifies the performance using three key metrics: Throughput (in Mbps), Bit Error Rate (BER), and Quality of Experience (QoE). The comparison is done across different user sizes: 50, 100, and 150.

The proposed Tasmanian Devil Whale Optimization (TDWO) algorithm demonstrates significantly superior performance compared to conventional resource allocation methods such as ARA, Network Resource Allocator, HEVC, and SVC in 5G networks for secure educational video transmission. Simulation results for various user sizes (50, 100, and 150) consistently show that TDWO achieves the highest throughput, lowest bit error rate (BER), and best Quality of Experience (QoE) among all evaluated approaches. These advantages indicate that TDWO, through optimized resource allocation and rapid convergence, not only enhances the reliability and efficiency of video transmissions but also improves user experience under different 5G network conditions, making it an effective solution for secure video delivery.

## 9 Conclusion

Multimedia communications are highly applied in the educational industry due to the rapid growth of internet data service transmission as well as communication technologies. At present, different educational industries highly utilize video applications for remote, efficient, and intuitive video communication. This research presents an effective resource allocation algorithm named TDWO for secure transmission of video in 5G networks. The input recorded video taken from the database is initially allowed for secure video transmission over 5G cellular networks. The allocation of resources in 5G cellular networks is performed via the designed TDWO resource allocation scheme. Moreover, fitness parameters such as the data rate, achievable data rate, and QoE are considered when allocating resources via TDWO. The QoE is effectively predicted by using the DCNN model in resource allocation. In addition, the results obtained from the experiment revealed the potential of TDWO in resource allocation for video transmission. The TDWO attained a throughput, BER, and QoE and a fitness of 25.557 Mbps, 0.021, 18.332 and 0.013, respectively. In the future, this research will be extended by devising lightweight models to increase the resource allocation efficiency in 5G networks.
